# Conjoined twins in a spontaneous monochorionic triplet pregnancy: A case report and literature review

**DOI:** 10.1097/MD.0000000000024490

**Published:** 2021-01-29

**Authors:** Qianqian Gao, Houqing Pang, Hong Luo

**Affiliations:** a Department of Ultrasound, West China Second University Hospital; b Key Laboratory of Obstetric & Gynecological and Pediatric Diseases and Birth Defects of Ministry of Education, Sichuan University, Chengdu, Sichuan, P.R. China.

**Keywords:** conjoined twins, literature review, omphalopagus, triplet pregnancy

## Abstract

**Rationale:**

Conjoined twins are a rare complication of monochorionic pregnancies and an extremely rare condition in spontaneous triplet pregnancies. We report a case of conjoined twins in a spontaneous monochorionic triplet pregnancy. The conjoined twins might have suffered from twin anemia-polycythemia sequence, which was reported to be extremely rare.

**Patient concerns:**

A 26-year-old woman conceived spontaneously with an obstetric history of invasive mole 4 years ago.

**Diagnoses:**

We initially misdiagnosed her as having monochorionic triamniotic triplets at 10 weeks of gestation. However, we confirmed conjoint twins with the monochorionic diamniotic triplet pregnancy at 12 weeks of gestation and classified them as omphalopagus.

**Interventions:**

As the woman decided to continue the pregnancy, regular and careful antenatal care was conducted.

**Outcomes:**

Unexpectedly, she had a stillbirth 3 weeks later and had to terminate the pregnancy at 15 weeks of gestation. After abortion, the diagnosis of omphalopagus was confirmed in the induced fetuses. Moreover, the skin colors of the conjoined twins were different: one was plethoric, and the other was pale. Additionally, the parents agreed to examine the chromosome of the fetuses, and the results were normal.

**Conclusion:**

Dichorionic triplet and monochorionic triplet pregnancies have a poorer prognosis than trichorionic triplet pregnancies. Surgery is the main therapy for conjoined twins; however, most conjoined twins in triplet pregnancies cannot survive, including omphalopagus twins. The conjoined twins may have suffered from twin anemia-polycythemia sequence, which could probably not be diagnosed intrauterine. Transvaginal probe and 3-dimensional ultrasound may be helpful for clarifying the diagnosis in early pregnancy.

## 1 Introduction

The incidences of twin and triplet pregnancies have increased gradually in recent years.^[1–3]^ Conjoined twins are a complication of multiple pregnancies, and the total prevalence of conjoined twins is approximately 1.47 per 100,000 births.^[4]^ It is extremely rare for conjoined twins to occur in a triplet pregnancy, with an incidence of nearly less than 1 in a million deliveries.^[5]^ The classification of conjoined twins includes ventral union, dorsal union, and lateral union.^[6]^ In the category of ventral union, cephalopagus, thoracopagus, omphalopagus, and ischiopagus twins could be diagnosed.^[6]^ The purpose of this report and review is to describe the presentation, management, and outcome of conjoined twins in triplet pregnancy, especially to evaluate the prognosis of omphalopagus twins over the last 20 years.

## 2 Case report

**Figure 1:**
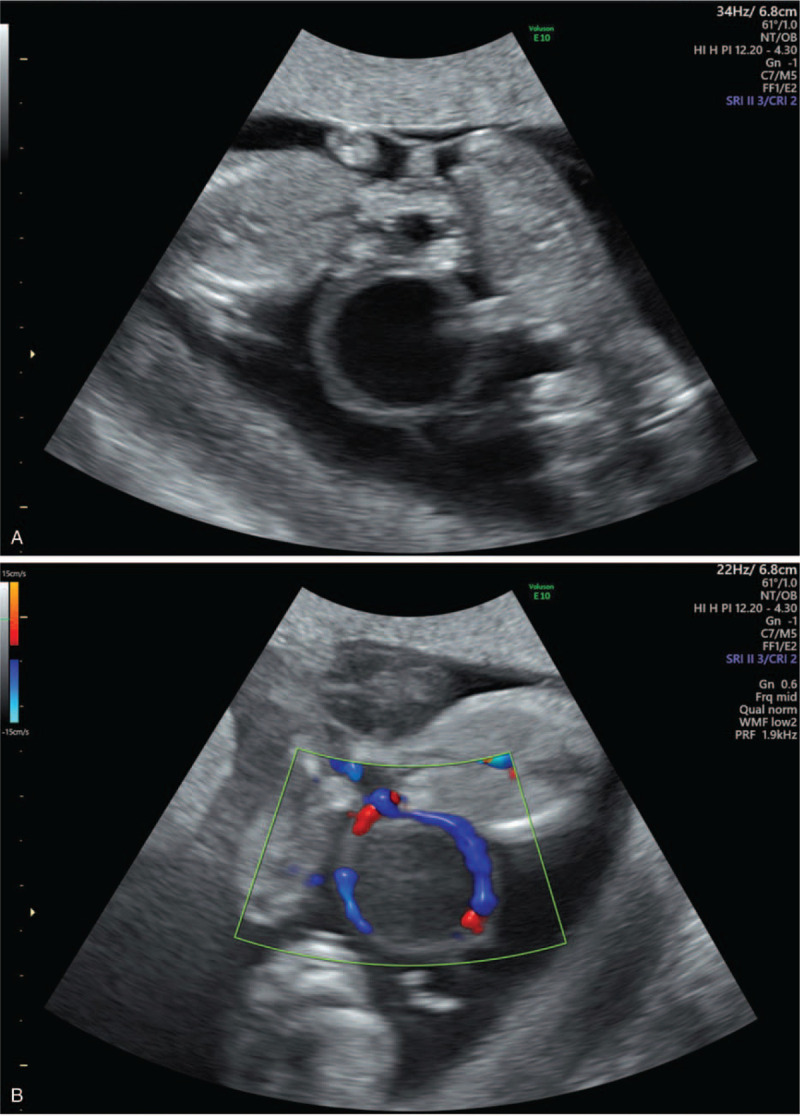
Two-dimensional ultrasound image (A) and color Doppler flow imaging (B) of the conjoined twins at 12 wk of gestation. The conjoined twins fused through the lower abdomen with a cystic structure.

**Figure 2:**
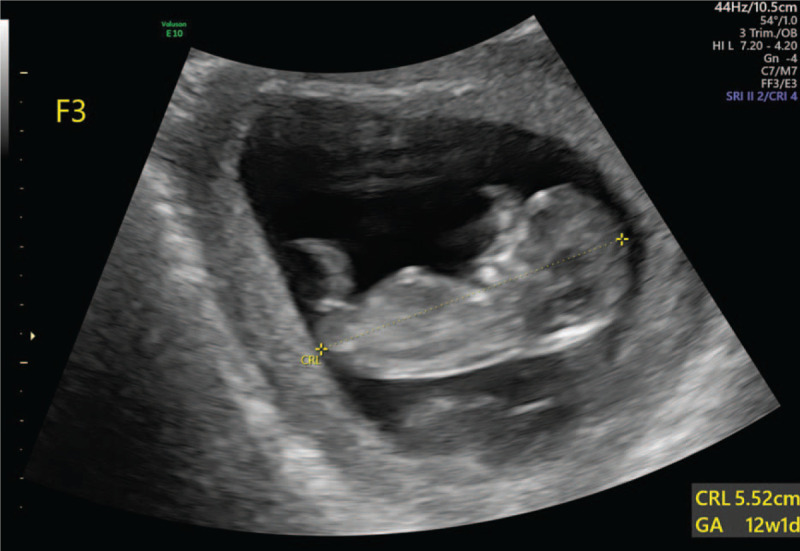
Two-dimensional ultrasound image of the normal nonconjoined fetus.

**Figure 3:**
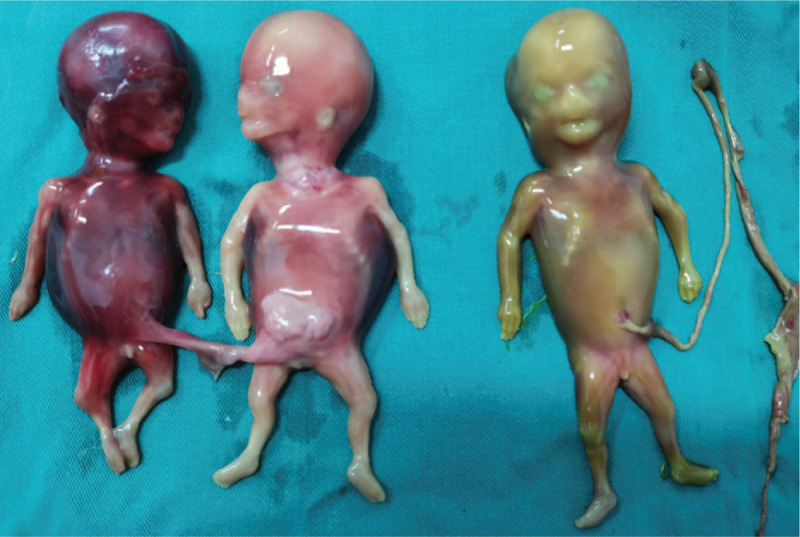
The postnatal anterior view of the triplet pregnancy. The conjoined twins were connected through a short umbilical cord; one was plethoric, and the other was pale.

A 26-year-old Chinese woman (gestation 2, parity 0, abortion 1) who spontaneously conceived presented to West China Second University Hospital. She had an obstetric history of abortion 4 years ago, when she was diagnosed with invasive mole and underwent 6 cycles of chemotherapy. Her past medical, surgical, and family history did not reveal any other problems. She spontaneously conceived at 10 weeks of gestation by the last menstrual period and presented to the Ultrasound Department for routine prenatal care. According to the transabdominal ultrasound, a triplet pregnancy was diagnosed at 10 weeks of gestation, and there was an amniotic sac between each fetus. Two weeks later, the woman underwent early pregnancy screening by sonography at 12 weeks of gestation. The woman was diagnosed with a MCDA triplet pregnancy by using 2-dimensional ultrasonography with a GE Voluson E10 ultrasound system (FL). One of the amniotic sacs contained a fetus with a crown-rump length of 5.52 cm. In the other amniotic sac, an antenatal diagnosis of conjoined twins in a triplet pregnancy was made. The crown-rump length of the conjoined twins were 4.63 cm and 4.89 cm. The conjoined twins fused through the lower abdomen with 2 separate heads, 2 hearts, 2 livers, 4 arms, 4 legs, and a single umbilical cord, which was diagnosed as omphalopagus (Fig. 1A and B). The conjoined twins had a short umbilical cord, abnormal lower abdomens, abnormal bladders, and a cystic structure between the pair. The detailed transabdominal ultrasound scan showed that the twins seemed to be joined with tissues such as bladder tissues, umbilical vessels, and a cyst connected with the bladder, which was surrounded by umbilical vessels (Fig. 1A and B). The structures of the single fetus did not show any abnormalities (Fig. 2). The nuchal translucency measurements of the 3 fetuses were normal (1.2 mm, 0.7 mm, and 1.0 mm). In the assessment of blood flow, the ductus venosus flow of 1 conjoined twin disappeared, and the other 2 were normal. After extensive counseling by the prenatal diagnosis doctor, the parents decided to continue the pregnancy. A transabdominal ultrasound scan was performed 20 days later; however, unexplained intrauterine demise of the 3 fetuses was observed. The pregnancy was terminated at 15 weeks of gestation by induced abortion at the hospital, and we obtained images of the induced fetuses, which confirmed the ultrasound diagnosis (Fig. 3). After abortion, we found that the skin colors of each fetus were different, especially of the conjoined twins. One of the conjoined twins was plethoric and the other was pale which might suffer from twin anemia-polycythemia sequence (TAPS). However, as the 3 fetuses died, we could not evaluate the hemoglobin levels. In addition, the 3 fetuses all underwent genetic tests, and the results were normal with 46 XY. Written informed consent was obtained from the patient for publication of the case details and accompanying images. In addition, the study was approved by the ethics committee of West China Second Hospital of Sichuan University.

## 3 Discussion

The incidence of conjoined twins in China is similar to that in Europe and America but lower than that in South America.^[4]^ It was reported that the incidence of twin and triplet pregnancies was higher than that 2 decades ago, which might be due to the application of assisted reproductive technology.^[7,8]^ However, few studies have reported conjoined twins in triplet pregnancies.^[5,9–11]^ We reviewed the literature about this topic over the last 20 years, and we found that nearly 73% of families chose to terminate the pregnancy or reduce the number of conjoined twins, but only 13% had a chromosome test of the fetuses performed.^[5,9,10,12–14]^ Although 4 families had chosen to continue the pregnancy, all the conjoined twins died intrauterine or after birth, and the single fetus survived.^[13,15–17]^ In this case, all fetuses died intrauterine. Detailed information on conjoined twins in triplet pregnancies is summarized in Table 1.

**Table 1:** Clinical characteristics of selected trials about conjoined twins in a triplet pregnancy.

							Outcome of the 3 fetuses		Fetal sex/Gene test	
Reference	Age	Mode of conception	Placentation	Gestational age at diagnosis (wk)	Type of conjoining	Management	Conjoined twins	Single fetus	Conjoined twins	Single fetus
Wax et al, 1999^[33]^	18	Spontaneous	MCMA	16 + 5	Thoracopagus	TOP	IUD	IUD	46, XX	46, XX
Goldberg et al, 2000^[14]^	28	ICSI	DCDA	8 + 4	Thoraco-omphalopagus	Selective fetal reduction at 12 wk	IUD	Live birth at 37 wk	Not clarified	Male
Zeng et al, 2002^[15]^	22	Spontaneous	MCDA	22	Thoracopagus	Expectant	Live birth at 32 wk, but died 35 d later	Live birth at 32 wk	Female	Female
Sepulveda et al, 2003^[16]^	41	Spontaneous	MCDA	13	Cephalopagus	Selective fetal reduction at 16 wk	IUD	IUD at 28 wk	Not clarified	Not clarified
	29	Spontaneous	DCDA	10^+3^	Thoracopagus	Expectant	IUD at 12 wk	Live birth at 38 wk	Not clarified	Not clarified
Charles et al, 2005^[22]^	Not clarified	ICSI	DCDA	10	Omphalopagus/ischiopagus/“Diamniotic vitellopagus”	Selective fetal reduction at 15 wk	IUD	PROM at 21 wk	Not clarified	Not clarified
Suzumori et al, 2006^[34]^	33	Spontaneous	MCDA	13	Cephalopagus	TOP	IUD	IUD	Female	Not clarified
Hirata et al, 2009^[17]^	34	ICSI	DCDA	8	Thoracopagus	Expectant	IUD at 10^+3^ wk	Live birth at 39 wk	Not clarified	Not clarified
Shepherd et al, 2011^[35]^	32	Clomiphene citrate	DCDA	13^+1^	Thoraco-omphalopagus	Selective fetal reduction at 13^+5^ wk	IUD	Live birth at 40 wk	Not clarified	Male
Sellami et al, 2013^[11]^	20	Spontaneous	MCDA	21	Xipho-omphalopagus	TOP	IUD	IUD	Not clarified	Female
Kaveh et al, 2014^[13]^	27	Spontaneous	Not clarified	15	Dicephalic parapagus tribrachius	Expectant	Live birth at 36 wk but died after 5 d	Live birth at 36 wk	Male	Male
Talebian et al, 2015^[10]^	38	ICSI	MCDA	12^+2^	Thoraco-omphalopagus	Selective fetal reduction at 16 wk	IUD	PROM at 17 wk	Not clarified	Not clarified
Ozcan et al, 2017^[9]^	28	Spontaneous	DCDA	17	Thoraco-omphalopagus	Selective fetal reduction at 21 wk	IUD	IUD at 21^+1^ wk	Male	Male
Yuan et al, 2017^[12]^	39	ICSI	MCDA	10	Thoraco-omphalopagus	TOP	IUD	IUD	46, XX	46, XX
Meng et al, 2018^[5]^	36	Spontaneous	MCDA	13^+5^	Thoraco-omphalopagus	Selective fetal reduction at 16 wk	IUD	IUD at 17 wk	Not clarified	Not clarified
Present case	26	Spontaneous	MCDA	12	Omphalopagus	Expectant	IUD at 15 wk	IUD at 15 wk	46, XY	46, XY

DCDA = dichorionic diamniotic, ICSI = intracytoplasmic sperm injection, IUD = intrauterine death, MCDA = monochorionic diamniotic, MCMA = monochorionic monoamniotic, PROM = premature rupture of membrane, TOP = termination of pregnancy.

According to studies on triplet pregnancy, Curado et al found that the risk of neurological morbidity was significantly higher in dichorionic triamniotic triplets than in trichorionic triamniotic triplets.^[18]^ In addition, the risk of perinatal death and the abortion rate were higher in Dichorionic triplet (DCT) pregnancies than in trichorionic triplet pregnancies, mainly owing to the higher risk of intrauterine death in DCT triplet pregnancies.^[18–20]^ Due to the small sample size of monochorionic triplet (MCT) cases, only a few researchers have studied MCT pregnancies and found that the outcomes of these pregnancies were poor, which may be due to monochorionic placentation.^[19,20]^ The clinical study was performed in accordance with our patient.

We also reviewed the literature about conjoined twins further, and most researchers considered the conjoined twins to belong to monoamniotic pregnancy.^[4,21]^ However, Adrian^[22]^ and Spencer^[23]^ noted that some minimally united twins with conjoined cloacae, which may be called “diamniotic minimally conjoined twins,” were omphalopagus, and the prevalence rate was 5.5% in conjoined twins.^[22,24,25]^ In our case, the characteristics of the conjoined twins were very similar to their reports. A detailed ultrasound scan revealed that the conjoined twins shared part of the abdominal wall, genitourinary system, and umbilical cord.^[25,26]^ Compared with other classifications of conjoined twins, omphalopagus twins have more opportunity to survive if they undergo successful surgery.^[25,26]^ The etiology of fetal death was probably due to hemodynamic instability.^[25]^ In our case, the intrauterine demise was probably due to TAPS, which is usually not diagnosed until after birth.^[27]^ For the early diagnosis of conjoined twins, researchers have tried to achieve a diagnosis with 3-dimensional ultrasound (3DUS) and transvaginal ultrasound and found that the prenatal assessment of conjoined twins with 3DUS was important for emergency postnatal surgical separation and early confirmation.^[28–30]^ According to the literature, we also found that most of the conjoined twins were female and not associated with genetic factors.^[4]^ For our patient, all 3 fetuses had normal chromosomes, but they were male.

In our report, we misdiagnosed the conjoined twins in a triplet pregnancy. First, we recognized amniotic sacs between each fetus that might be caused by the early gestational week, the low joined part of the twins and the resolution of transabdominal ultrasound. After the diagnosis of conjoined twins, we tried to use 3DUS to distinguish the details, but we did not obtain the expected images as reported in the literature.^[31,32]^ Unfortunately, we did not perform vaginal ultrasound in the patient at the first examination, which might have improved the resolution and helped us diagnose conjoined twins earlier.

## 4 Conclusion

In conclusion, DCT pregnancies have a higher risk of perinatal death and higher abortion rates than trichorionic triplet pregnancies, but MCT pregnancies have the worst prognosis. Although omphalopagus twins have a greater chance of surviving, most conjoined twins in triplet pregnancies could not survive, including our patient, which may be due to TAPS. In the future, we will try to use transvaginal ultrasound and 3DUS to improve the resolution for diagnosis in early pregnancy. The sooner the diagnosis of conjoined twins is made, the better the counseling and therapy.

## Author contributions

**Conceptualization:** Houqing Pang.

**Investigation:** Qian Qian Gao.

**Methodology:** Hong Luo.

**Project administration:** Houqing Pang.

**Supervision:** Houqing Pang.

**Writing – original draft:** Qian Qian Gao.

**Writing – review & editing:** Qian Qian Gao, Houqing Pang.

## Abbreviations

Abbreviations: 3DUS = 3-dimensional ultrasound, DCT = dichorionic triplet, MCDA = monochorionic diamniotic, MCT = monochorionic triplet, TAPS = twin anemia-polycythemia sequence.
